# Quality of life and factors associated with a good quality of life among diabetes mellitus patients in northern Thailand

**DOI:** 10.1186/s12955-022-01986-y

**Published:** 2022-05-20

**Authors:** Ratipark Tamornpark, Suphaphorn Utsaha, Tawatchai Apidechkul, Dunlayaphap Panklang, Fartima Yeemard, Peeradone Srichan

**Affiliations:** 1 School of Health Science, Mae Fah Luang University, 333 Moo 1, Ta Sud Subdistrict, Muang District, 57100 Chiang Rai Province Thailand; 2 Center of Excellence for the Hill Tribe Health Research, Mae Fah Luang University, Chiang Rai, 57100 Thailand; 3 Wiang Chai District Public Health Office, Chiang Rai, 57210 Thailand

**Keywords:** Quality of life, Diabetes mellitus, Factors associated, COVID-19

## Abstract

**Background:**

Quality of life (QOL) is a good indicator of lifespan, especially for individuals who are suffering from a particular illness. QOL among patients with diabetes mellitus (DM) could be used for further implementations in addition to improving patient care and disease management, especially during the coronavirus disease 2019 (COVID-19) pandemic. This study aimed to assess QOL and identify factors associated with a good QOL among DM patients in northern Thailand.

**Methods:**

A cross-sectional study was conducted to gather information from DM patients attending six randomly selected hospitals in the Chiang Rai province, northern Thailand. A validated questionnaire and the 26-item quality of life brief version (WHOQOL-BREF) were used to collect socioeconomic factors and assess QOL, respectively. Chi-square tests and logistic regression were used to detect the associations between variables at a significance level of α = 0.05.

**Results:**

A total of 967 participants were enrolled in the study: 58.8% were female, 52.3% were aged ≥ 60 years, 79.7% graduated primary school and had no additional education, 68.7% had an annual income ≤ 50,000 baht, and 29.3% were unemployed. The majority of patients had a poor-to-moderate overall QOL (49.4%); 90.1% reported a moderate QOL in the physical domain, 54.7% reported a moderate QOL in the mental domain, 63.4% reported a good QOL in the social relationship domain, and 50.6% reported a good QOL in the environmental domain. In multivariate analysis, seven variables were found to be associated with good QOL among the participants. Those aged ≤ 59 years had 1.90 times (95% CI 1.32–2.73) greater odds of having good QOL than those aged ≥ 60. Those who had annual income ≥ 100,001 baht had 2.16 times (95% CI 1.17–3.96) greater odds of having good QOL than those who had annual income ≤ 50,000 baht. Those who lived alone and with spouses had 3.38 times (95% CI 1.42–8.02) and 2.20 times (95% CI 1.20–4.02) greater odds of having good QOL, respectively, than those who lived with relatives. Those who exercised regularly had 4.72 times (95% CI 2.71–8.19) greater odds of having good QOL than those who never exercised. Those who had a high level of knowledge regarding prevention and care had 3.26 times (95% CI 1.22–5.55) greater odds of having good QOL than those who had low knowledge. Those who did not have diabetic nephropathy had 7.41 times (95% CI 4.99–11.01) greater odds of having good QOL than those who were diagnosed with diabetic nephropathy, and those whose medical fees were supported by the government under the universal scheme had 4.31 times (95% CI 1.15–16.7) greater odds of having good QOL than those who had to support themselves.

**Conclusions:**

Almost a half of DM patients in northern Thailand reported having a low-to-moderate QOL, which can be improved by focusing on socioeconomic factors, family support as well as improving knowledge regarding DM prevention and care, including the support of medical fees.

**Supplementary Information:**

The online version contains supplementary material available at 10.1186/s12955-022-01986-y.

## Introduction

Quality of life (QOL) is an important aspect of human life and is related to the culture and values systems in which individuals live as well as their goals and expectations [1]. Generally, individuals who have an illness tend to have a poorer QOL than those who are free of disease or illness. It is difficult to improve an individual’s physical health without considering QOL, especially for those who are diagnosed with chronic noncommunicable diseases (NCDs). Diabetes mellitus (DM) is one of the most significant NCDs globally and is one of the major contributors to poorer QOL [2]. Many factors have been clearly identified as contributors to poorer QOL among DM patients, such as age [3], economic status [4], education [5], quality of medical care [6] and the complication stage of the disease [7]. Having a poorer QOL leads to several impacts on both mental and physical health among individuals with DM [8] and their family members [9].

QOL is always related to a population’s lifestyle and culture, and people living in northern Thailand have their own culture, especially with respect to dietary behaviors and socioeconomic status [10]. In regards to dietary behaviors, people in northern Thailand tend to favor eating sticky rice and oily noodles [11, 12]. Moreover, for years, northern Thailand has had the largest proportion of people aged 60 years and over [13]. The majority of people work in agricultural sectors and have poor opportunities to access schools. People residing in this area are vulnerable to many health problems, including DM. Since the first detected case of coronavirus disease-2019 (COVID-19) in Thailand in March 2020 [14], all medical and public health services provided by public hospitals have been modified with the goal of COVID-19 prevention and control, including the frequency of attending the clinic to measure blood glucose and case management of DM patients; the time between these visits has been extended 3–6 months each time [15]. The lower frequency of monitoring blood glucose levels among DM patients living in northern Thailand, with its unique culture, may impact the QOL of these patients.

Since the beginning of the COVID-19 pandemic, all medical and public health services in Thailand have been modified to promote disease prevention and control measures, especially to reduce contact with suspected cases in hospitals and to maintain social distancing [15]. By implementing these measures, many hospitals have modified their schedule for DM case management, including blood glucose monitoring, which might impact the effectiveness of disease management and contribute to a poorer QOL. The World Health Organization Quality of Life Instrument, Short Form (WHOQOL-BREF) [1] was used to assess QOL among DM patients living in northern Thailand. The aims of the study were to determine the QOL among DM patients attending public hospitals in the northernmost section of Thailand after modifications were made to the schedule of blood glucose monitoring and case management during the COVID-19 pandemic and to identify the factors associated with a good QOL in these individuals. The findings could be used to improve the QOL of DM patients who are suffering from their physical pathogenesis.

## Methods

A cross-sectional study design was used to collect information from the participants. Participants included patients with DM attending 6 hospitals: Mae Chan Hospital, Mae Lao Hospital, Phan Hospital, Wiang Chiang Rung Hospital, Mae Lao Health Promoting Hospital, and Wiang Chai Health Promoting Hospital, which were selected by a simple random method from 18 local public hospitals in Chiang Rai Province, Thailand.

All patients with DM diagnosed at least two years prior to the study, aged ≥ 18 years and attending at one of six hospitals between February and May 2021 met the inclusion criteria and were invited to participate in the study. Those who were in the stage of severe illness, first diagnosis, unable to provide key information regarding the questionnaire, and pregnant women were excluded from the study.

The sample size was calculated based on the standard formula for a cross-sectional design [16]: n = [Z^2^_α/2_P(1 − P)]/e^2^, where Z^2^_α/2_ = 1.96, P = 0.39 [6], Q = 0.61, e = 0.03, and adding for 10.0% for any errors during the study; therefore, 953 participants were required for the analysis.

Validated questionnaires and 5-mL blood specimens were used as research instruments. The questionnaire was developed by the researcher, and questions were assessed for validity and reliability before use. To assess validity, the item-objective congruence (IOC) method was used. The IOC method allowed three experts in the field (two psychologists and one public health professional) to assess the QOL the questions and determine the relevance of the questions to the study objective. Each expert gave a score to each item, “−1” meant that the question was not related to the context of the study, “0” meant the question was related to the context of the study but required improvement before use, and “+1” meant that the question was well relevant to the context of the study. Afterward, the scores from experts were polled and averaged prior to interpretation. Questions that scored less than 0.5 were excluded from the questionnaire. The questions that scored between 0.51 and 0.70 were modified before inclusion in the questionnaire. Questions that scored greater than 0.7 were included in the questionnaire.

The questionnaire was then determined to be reliable among 25 participants who had similar characteristics to the study participants in Mae Chan district, Chiang Rai Province, Thailand. In this stage, feasibility, understandability, and sequence of the questions were assessed. The Cronbach alpha was found at 0.74 (Additional file 1).

Finally, seven parts of the questionnaire were used to collect data in the study. In part one, 7 open-ended questions assessed complete physical examinations and laboratory data, such as weight, height, blood pressure, HbA1c and lipid profiles. A glycated hemoglobin (HbA1c) level ≥ 7 was defined as uncontrolled blood glucose [17]. In part two, 20 questions were used to collect general information from the participants, such as sex, age, religion, education, and occupation. In part three, 8 questions were used to collect data on health behaviors such as smoking behavior, alcohol use, and tea or coffee consumption. In part four, the standard stress test (ST-5) [18] was used to assess stress levels: low (≤ 4 points), moderate (5–7 points), and high (≥ 8 points). The ST-5 was developed by the Department of Mental Health, Ministry of Public Health Thailand. In part five, 10 questions were used to assess the level of knowledge of DM prevention and care: those who scored < 6 were classified as having poor knowledge, individuals with scores between 6 and 7 were classified as having moderate knowledge, and individuals with scores ≥ 8 were classified as having a high level of knowledge. In part six, 10 questions were used to assess attitudes toward DM prevention and care: those who scored < 6 were classified as having negative attitudes, individual with scores between 6 and 7 were classified as having neutral attitudes, and individuals with scores ≥ 8 were classified as having positive attitudes. In the last part, the 26-item World Health Organization Quality of Life Instrument, Short Form (WHOQOL-BREF) [19] was used to assess the QOL among the participants in four domains: physical health, mental health, social relationships, and environment health. Question no. 2–4, 10–12, and 24 were used to detect physical health. Question no. 5–9, and 23 were used to detect mental health. Question no. 13, 14 and 25 were used to detect social relationships. Question no. 15–22 were used to detect the environmental health. Question no.1 and 26 were used to detect the overall quality of life. Each question was provided with five scales: 1, 2, 3, 4, 5 where 1 represents “disagree”, and 5 represents completely agree. The scores on the physical health were divided into 3 levels: poor (7–16 points), moderate (17–26 points), and good (27–35 points). The scores of the mental health were divided into 3 levels: poor (6–14 points), moderate (15–22 points), and good (23–30 points). The scores for the social relationships were divided into 3 levels: poor (3–7 points), moderate (8–11 points), and good (12–15 points). The scores of the environmental health were divided into 3 levels: poor (8–18 points), moderate (19–29 points), and good (30–40 points. The overall quality of life was divided into 3 levels: poor (26–60 points), moderate (61–95 points), and high (96–130 points). The WHOQOL-BREF in Thai form which was used in the study was completely developed by the WHO and freely available [20].

### Data gathering procedures

After obtaining the approval of the institutional review board (IRB) for conducting research in human subjects, selected hospital directors and the chief clinics were contacted and explained the study objectives and other relevant information. Once there was agreement to collect data, all DM patients who were attending and met the inclusion criteria were invited to participate in the study. All participants were asked to provide informed consent before interviews and blood specimen collection. For those who could not read the documents, the researcher read and explained all information to the participants and asked them to confirm their understanding of the whole context before requesting that the participants sign the informed consent form. The questionnaire was completed by researchers during interview. Each interview lasted for 20 min. Afterwards, 5 mL blood specimens were drawn and transferred to the Mae Fah Laung Medical Laboratory Center for laboratory work.

### Statistical analysis

Data were coded and cleaned before transferring into SPSS version 24, 2016 (SPSS, Chicago, IL) for analysis. All independent variables were categorized properly and checked for the completion before further analysis. Dependent variable was classified according to the levels of QOL. Descriptive statistics were used to describe the general characteristics of the participants. Continuous data with normal distributions are presented as the mean and SD. For continuous data with distribution in skewness form, the median and IQR are presented. Percentages were used to present all categorical data. Chi-square tests and logistic regression were used to detect the associations between variables at the significance level of α = 0.05. In the univariate and multivariate logistic regression models, two categories of QOL were classified as the dependent variable: poor-to-moderate, and good QOL. The mode of “ENTER” was used to select the variable into the model. The pseudo R^2^ and the Hosmer–Lemeshow chi-square were determined for fitting the final model.

## Results

A total of 967 participants were recruited into the study: 58.8% were female, 52.3% were aged ≥ 60 years (mean = 58.7, and SD = 11.3), and 78.5% were married. A large proportion graduated primary school and had no further education (79.7%), 68.7% had an annual income ≤ 50,000 baht, and 29.3% were unemployed (Table 1).

**Table 1:** General characteristics among the participants (n = 967)

Characteristics	n	%
*Sex*		
Male	398	41.2
Female	569	58.8
*Age* (years)		
≤ 59	461	47.7
≥ 60	506	52.3
Mean = 58.7, SD = 11.3		
*Marital status*		
Single	70	7.2
Married	759	78.5
Ever married	138	14.3
*Religion*		
Buddhism	965	99.5
Christian	5	0.5
*Education*		
No education	122	12.6
Primary school	649	67.1
≥ High school	196	20.3
*Occupation*		
Unemployed	283	29.3
Farmer	352	36.4
Employer	332	34.3
*Annual income* (baht)		
≤ 50,000	664	68.7
50,001–100,000	189	19.5
≥ 100,001	114	11.8
*Family members* (people)		
≤ 4	828	85.6
≥ 5	139	14.4
*Living with*		
Alone	61	6.3
Spouse	589	60.9
Child	216	22.3
Relatives	101	10.5
*Smoking*		
No	810	83.8
Yes	157	16.2
*Alcohol consumption*		
No	813	84.1
Yes	154	15.9
*Exercise*		
No	499	51.6
Sometimes	265	27.4
Regularly	203	21.0
*Stress* (ST-5)		
Low	719	74.4
Moderate	159	16.4
High	89	9.2
*Length of having diabetes* (year)		
≤ 10	709	73.3
11–20	172	17.8
> 20	86	8.9
*Controlled blood glucose*		
No	530	54.8
Yes	437	45.2
*Experience of having side effect of taking diabetes medicines*		
No	836	86.5
Yes	131	13.5
*Experience of having wound on foots*		
No	907	93.8
Yes	60	6.2
*Diabetic nephropathy*		
Yes	375	38.8
Do not know	486	59.3
No	106	11.0
*Having hypertension*		
Yes	395	40.8
Do not know	517	53.5
No	55	5.7
*Medical expenses*		
Under the national universal scheme	947	97.9
Self-payment	20	2.1
*Knowledge regarding DM prevention and care*		
Low	78	8.1
Moderate	384	39.7
High	505	52.2
*Attitudes regarding DM prevention and care*		
Poor	270	27.9
Moderate	562	58.1
Positive	135	14.0

Regarding health behaviors, 16.2% of participants smoked, 15.9% used alcohol, and 25.6% reported moderate-to-high stress. One-fourth of participants (26.7%) had been diagnosed with DM for greater than 10 years, 54.8% had uncontrolled blood glucose levels, 13.5% had experienced side effects from taking a medication, 38.8% had diabetic nephropathy, 40.8% had hypertension (HT) comorbidity, and only 2.1% used self-payment for DM care and treatment. A large proportion of participants had low-to-moderate knowledge (47.8%) and attitudes (86.0%) toward DM prevention and care (Table 1).

Regarding the WHOQOL-BREF scores, almost half of the participants (49.4%) had poor-to-moderate levels of overall QOL. The majority of the participants had moderate levels of QOL in the physical domain (90.1%) and mental domain (54.7%). In the social relationships and environmental domains, 63.4% and 50.6% of participants were indicated good levels of QOL, respectively. The mental (*p*-value = 0.005) and overall domains (*p*-value = 0.005) of QOL were found to be significantly different between the uncontrolled blood glucose and controlled blood glucose groups (Table 2).

**Table 2:** WHOQOL-BREF among the participants by uncontrolled and controlled blood glucoses

Domain	Total n (%)	Uncontrolled n (%)	Controlled n (%)	χ^2^	*p*-value
*Physical health*				2.34	0.309
Poor	81 (8.4)	38 (7.1)	43 (9.8)		
Moderate	871 (90.1)	483 (91.2)	388 (88.8)		
Good	15 (1.6)	9 (1.7)	6 (1.4)		
*Mental health*				10.45	0.005*
Poor	34 (3.5)	18 (3.4)	16 (3.6)		
Moderate	529 (54.7)	266 (50.2)	263 (60.2)		
Good	404 (41.8)	246 (46.4)	158 (36.2)		
*Social relationships*				4.56	0.102
Poor	63 (6.5)	30 (5.7)	33 (7.5)		
Moderate	336 (34.7)	173 (32.6)	163 (37.3)		
Good	568 (58.7)	327 (61.7)	241 (55.2)		
*Environment health*				4.09	0.129
Poor	19 (2.0)	10 (1.9)	9 (2.1)		
Moderate	335 (34.6)	169 (31.9)	166 (38.0)		
Good	613 (63.4)	351 (66.2)	262 (59.9)		
*Overall*				9.72	0.005*^a^
Poor	5 (0.5)	4 (0.8)	1 (0.3)		
Moderate	473 (48.9)	236 (44.5)	237 (54.2)		
Good	489 (50.6)	290 (54.7)	199 (45.5)		

*Significant level at α = 0.05

^a^Fisher’s exact test

When comparing the QOL of uncontrolled and controlled blood glucose groups by sex, the mental health domain in males and the social relationships domain in females were found to be significantly different (Table 3).

**Table 3:** WHOQOL-BREF by sex

Sex	Domain	Group	WHOQOL-BREF			χ^2^	*p*-value
			Poor (%)	Moderate (%)	Good (%)		
Male	Physical health	Uncontrolled	13 (6.5)	183 (92.0)	3 (1.5)	1.62	0.445
		Controlled	20 (10.1)	176 (88.4)	3 (1.5)		
	Mental health	Uncontrolled	3 (1.5)	96 (48.2)	100 (50.3)	6.38	0.041*^,a^
		Controlled	4 (2.0)	120 (60.3)	75 (37.7)		
	Social relationships	Uncontrolled	5 (2.5)	66 (33.2)	128 (64.3)	5.26	0.072
		Controlled	15 (7.5)	63 (31.7)	121 (60.8)		
	Environment health	Uncontrolled	3 (1.5)	57 (28.6)	139 (69.9)	3.21	0.200
		Controlled	4 (2.0)	73 (36.7)	122 (61.3)		
Female	Physical health	Uncontrolled	25 (7.6)	300 (90.6)	6 (1.8)	1.03	0.616
		Controlled	23 (9.6)	212 (89.1)	3 (1.3)		
	Mental health	Uncontrolled	15 (4.5)	170 (51.4)	146 (44.1)	4.92	0.088
		Controlled	12 (5.0)	143 (60.1)	83 (34.9)		
	Social relationships	Uncontrolled	25 (7.6)	107 (32.3)	199 (60.1)	5.89	0.050*
		Controlled	18 (7.6)	100 (42.0)	120 (50.4)		
	Environment health	Uncontrolled	7 (2.1)	112 (33.8)	212 (64.1)	1.66	0.418
		Controlled	5 (2.1)	93 (39.1)	140 (58.8)		

*Significant level at α = 0.05

^a^Fisher’s exact test

Considering the QOL of uncontrolled and controlled blood glucose groups by age category, the physical and mental health domains were found to be significantly different among individuals aged ≤ 59. However, there were no significant differences between domains among individuals aged ≥ 60 (Table 4).

**Table 4:** WHOQOL-BREF by age category

Age (years)	Factor	DM group	WHOQOL-BREF			χ^2^	*p*-value
			Poor (%)	Moderate (%)	Good (%)		
≤ 59	Physical health	Uncontrolled	15 (5.3)	266 (93.6)	3 (1.1)	10.70	0.004*^a^
		Controlled	24 (13.6)	149 (84.2)	4 (2.2)		
	Mental health	Uncontrolled	14 (4.9)	118 (41.6)	152 (53.5)	8.56	0.014*
		Controlled	4 (2.3)	97 (54.8)	76 (42.9)		
	Social relationships	Uncontrolled	20 (7.1)	81 (28.5)	183 (64.4)	0.19	0.906
		Controlled	14 (7.9)	52 (29.4)	111 (62.7)		
	Environment health	Uncontrolled	9 (3.2)	78 (27.4)	197 (69.4)	1.14	0.595
		Controlled	4 (2.3)	56 (31.6)	117 (66.1)		
≥ 60	Physical health	Uncontrolled	23 (9.4)	217 (88.2)	6 (2.4)	2.95	0.217^a^
		Controlled	19 (7.3)	239 (91.9)	2 (0.8)		
	Mental health	Uncontrolled	4 (1.6)	148 (60.2)	94 (38.2)	5.46	0.064
		Controlled	12 (4.6)	166 (63.8)	82 (31.6)		
	Social relationships	Uncontrolled	10 (4.1)	92 (37.4)	144 (58.5)	4.90	0.090
		Controlled	19 (7.3)	111 (42.7)	130 (50.0)		
	Environment health	Uncontrolled	1 (0.4)	91 (37.0)	154 (62.6)	4.35	0.108^a^
		Controlled	5 (1.9)	110 (42.3)	145 (55.8)		

*Significant level at α = 0.05

^a^Fisher’s exact test

Univariate logistic regression was used to identify factors associated with WHOQOL-BREF scores among the DM patients. All variables were found to be associated with QOL: age, sex, education, occupation, annual income, number of family members the individual is living with, smoking, alcohol use, exercise, stress, knowledge regarding DM prevention and control, attitudes toward DM prevention and control, time since DM diagnosis, presence of side effects from taking DM medicine, presence of wounds on feet, diabetic nephropathy, having HT, and coverage of medical expenses (Table 5).

**Table 5:** Factors associated with a good QOL in univariate and multivariate analyses

Factors	QOL		Univariate analysis			Multivariate analysis		
	Good n (%)	Poor-moderate n (%)	OR	95% CI	*p*-value	AOR	95% CI	*p*-value
*Sex*								
Male	217 (54.5)	181 (45.5)	1.30	1.01–1.69	0.040*			
Female	272 (47.8)	297 (52.2)	1.00					
*Age* (years)								
≤ 59	272 (59.0)	189 (41.0)	1.91	1.48–2.47	< 0.001*	1.90	1.32–2.73	< 0.001*
≥ 60	217 (42.9)	289 (57.1)	1.00			1.00		
*Education*								
No education	42 (34.4)	80 (65.6)	1.00					
Primary school	338 (52.1)	311 (47.9)	2.07	1.38–3.10	< 0.001*			
≥ High school	109 (55.6)	87 (44.4)	2.38	1.49–3.81	< 0.001*			
*Occupation*								
Unemployed	139 (49.1)	144 (50.9)	1.00					
Agriculturist	167 (47.4)	185 (52.6)	0.93	0.68–1.27	0.675			
Trader	66 (68.7)	30 (31.3)	2.27	1.39–3.72	0.001*			
Employed	117 (49.6)	119 (50.4)	1.01	0.72–1.43	0.917			
*Annual income* (baht)								
≤ 50,000	294 (44.3)	370 (55.7)	1.00			1.00		
50,001–100,000	107 (56.6)	82 (43.4)	1.64	1.18–2.27	0.003*	0.96	0.62–1.48	0.867
≥ 100,001	88 (77.2)	26 (22.8)	4.26	2.68–6.77	< 0.001*	2.16	1.17–3.96	0.001*
*Number of family members* (people)								
≤ 4	403 (48.7)	425 (51.3)	1.00					
≥ 5	86 (61.9)	53 (38.1)	1.71	1.18–2.47	0.004*			
*Living with*								
Alone	38 (62.3)	23 (37.7)	3.25	1.67–6.31	< 0.001*	3.38	1.42–8.02	0.006*
Spouse	337 (57.2)	252 (42.8)	2.63	1.69–4.10	< 0.001*	2.20	1.20–4.02	0.010*
Child	80 (37.0)	136 (63.0)	1.15	0.70–1.90	0.560	0.69	0.35–1.36	
Relatives	34 (33.7)	67 (66.3)	1.00			1.00		
*Smoking*								
No	368 (45.4)	442 (54.6)	1.00					
Yes	121 (77.1)	36 (22.9)	4.03	2.71–6.00	< 0.001*			
*Alcohol use*								
No	381 (46.9)	432 (53.1)	1.00					
Yes	108 (70.1)	46 (29.9)	2.66	1.83–3.86	< 0.001*			
*Exercise*								
No	191 (38.3)	308 (61.7)	1.00			1.00		
Sometimes	130 (49.1)	135 (50.9)	1.55	1.14–2.09	0.004*	1.28	0.87–1.87	0.200
Regularly	168 (82.8)	35 (17.2)	7.74	5.15–11.62	< 0.001*	4.72	2.71–8.19	< 0.001*
*Stress* (ST-5)								
Low	388 (54.0)	331 (46.0)	0.62	0.39–0.99	0.046*			
Moderate	43 (27.0)	116 (73.0)	0.19	0.11–0.34	< 0.001*			
High	58 (65.2)	31 (34.8)	1.00					
*Knowledge regarding DM prevention and care*								
Low	17 (21.8)	61 (78.2)	1.00			1.00		
Moderate	117 (30.5)	267 (69.5)	1.57	0.88–2.80	0.126	1.33	0.65–2.72	0.430
High	355 (70.3)	150 (29.7)	8.49	4.80–15.02	< 0.001*	3.26	1.22–5.55	0.001*
*Attitudes regarding DM prevention and care*								
Poor	95 (35.2)	175 (64.8)	1.00					
Moderate	294 (52.3)	268 (47.7)	2.02	1.49–2.72	< 0.001*			
Positive	100 (74.1)	35 (25.9)	5.26	3.32–8.32	< 0.001*			
*Length of having DM* (year)								
≤ 10	348 (49.1)	361 (50.9)	1.71	1.07–2.72	0.023*			
11–20	110 (64.0)	62 (36.0)	3.14	1.83–5.39	< 0.001*			
> 20	31 (36.0)	55 (64.0)	1.00					
*Experience on having side effect from taking DM medicine*								
No	375 (44.9)	461 (55.1)	1.00					
Yes	114 (87.0)	17 (13.0)	8.24	4.86–13.97	< 0.001*			
*Experience on having wound on foots*								
No	439 (48.4)	468 (51.6)	1.00					
Yes	50 (83.3)	10 (16.7)	5.33	2.67–10.64	< 0.001*			
*Diabetic nephropathy*								
Yes	126 (25.9)	360 (74.1)	1.00			1.00		
Do not know	88 (83.0)	18 (17.0)	13.96	8.09–24.11	< 0.001*	2.36	0.96–5.27	0.055
No	275 (73.3)	100 (26.7)	7.85	5.78–10.66	< 0.001*	7.41	4.99–11.01	< 0.001*
*Controlled blood glucose*								
Yes	199 (45.5)	238 (54.5)	1.00					
No	290 (54.7)	240 (45.3)	1.44	1.12–1.86	0.005*			
*Having hypertension*								
Yes	245 (47.4)	272 (52.6)	1.00					
No	194 (49.1)	201 (50.9)	1.07	0.82–1.39	0.605			
Do not know	50 (90.9)	5 (9.1)	11.10	4.35–28.29	< 0.001*			
*Medical expenses*								
Pay by self	15 (75.0)	5 (25.0)	1.00			1.00		
Under the universal scheme	474 (50.1)	473 (49.9)	0.33	0.12–0.92	0.035*	4.31	1.15–16.17	0.030*

*Significance level α = 0.05

In multivariate analysis, seven variables were found to be associated with good WHOQOL-BREF scores among the participants: age, annual income, living situation, exercise, knowledge regarding DM prevention and control, diabetic nephropathy, and coverage of medical expenses. Those aged ≤ 59 years had 1.90 times (95% CI 1.32–2.73) greater odds of having good QOL than those aged ≥ 60. Those who had annual income ≥ 100,001 baht had 2.16 times (95% CI 1.17–3.96) greater odds of having good QOL than those who had annual income ≤ 50,000 baht. Those who lived alone and with spouses had 3.38 times (95% CI 1.42–8.02) and 2.20 times (95% CI 1.20–4.02) greater odds of having good QOL, respectively, than those who lived with relatives. Those who exercised regularly had 4.72 times (95% CI 2.71–8.19) greater odds of having good QOL than those who never exercised. Those who had a high level of knowledge regarding prevention and care had 3.26 times (95% CI 1.22–5.55) greater odds of having good QOL than those who had low knowledge. Those who did not have diabetic nephropathy had 7.41 times (95% CI 4.99–11.01) greater odds of having good QOL than those who were diagnosed with diabetic nephropathy, and those whose medical fees were supported by the government under the universal scheme had 4.31 times (95% CI 1.15–16.7) greater odds of having QOL than those who had to support themselves (Table 5).

## Discussion

Diabetes patients attending hospitals in northern Thailand had low-to-moderate levels of QOL in the physical and mental domains, while they had good levels of WHOQOL-BREF score in the social relationship and environmental domains. The mental and overall domains were different between those who were able to control and those who could not control their blood glucose. Several socioeconomic factors, lifestyle factors, and knowledge regarding DM prevention and care, including financial support for the burden of medical fees, were found to be associated with having a high WHOQOL-BREF score.

A study in Brazil reported that QOL among DM patients was good in the social relationship and phycological domains [21]. Godman et al. [22] reported that the physical and mental domains of QOL were significantly poorer among DM patients in Botswana. A study conducted in Indonesia reported that QOL among DM patients was detected at a good level in three domains: physical health, social relationships, and environmental health [23]. Another study conducted among Asian patients with type 2 DM reported that the QOL among DM patients was poor, especially those who could not control their blood glucose [24]. On the other hand, a study among DM patients living in Central Thailand in 2019 showed that more than half of DM patients had good QOL [6]. Khunkaew et al. [25] reported that the overall QOL among patients with type 2 DM living in northern Thailand was poor.

Our study found that DM patients aged 59 years and younger had better QOL than those aged 60 years and over, which is consistent with a study conducted in India that reported that QOL among DM patients was significantly reduced as age of patients increased [26]. A study in Iran that assessed QOL among DM patients using the EQ-5D-5L also found that those who were younger had a better QOL [27, 28]. Similarly, a study in Botswana reported that those who were older were at the poorer stage of physical domain in their QOL compared to those younger age [23]. A study in Palestine also reported that those who were living with DM for a longer time were associated with a more significant negative impact on QOL [29]. Younger patients with a better QOL could have a better chance to gain income from their job and are also more able to meet people in daily life, which directly supports both physical and mental health.

Diabetes mellitus patients living in northern Thailand with poor family economic status had poorer QOL than those living with higher family income. This was supported by a study conducted in Iran that reported that those with type 2 DM with lower income had poorer QOL [28]. Mngomezulu et al. [30] also reported that those with type 2 DM living with low family income had poorer QOL than those who were living with higher income families in Swaziland. Moreover, a study in Saudi Arabia reported that individuals with type 2 DM with higher family income had a significantly higher QOL than those living with lower incomes [31]. Finally, a study assessing QOL among DM patients living in Central Thailand in 2019 reported that social support, including family members and a high level of family income, was positively correlated with good QOL [32]. Having high income could directly support DM patients’ QOL, especially those living with high-income families, as they could be able to afford to seek medical services without financial barriers.

Regular exercise was shown to lead to good QOL among DM patients living in northern Thailand. This was confirmed by a systematic review study that reported that exercise could have a significantly positive impact QOL among type 2 DM patients [33]. Kueh et al. [34] also reported that self-management in terms of exercise and maintaining an exercise regimen predicted good QOL among DM patients in Australia. Moreover, a randomized controlled trial clearly showed that patients with type 2 diabetes who exercise had better QOL in the physical, psychological and environmental domains than those in the control group (non-exercising group) [35]. Colberg et al. [36] supported that exercise led to improved general health in type 2 DM patients and eventually improved QOL. Regular exercise could improve blood circulation and reduce the opportunity to have complications from the disease, which is one of the major contributing factors of poor QOL among DM patients [37].

Having a high knowledge of DM prevention and care was found to be one of the significant factors leading to good QOL among DM patients in northern Thailand. This was supported by a study conducted by Elazhary et al. [38], who reported that patients with type 2 DM who had a better knowledge of DM prevention and care had a better QOL than those who had a lower knowledge of DM prevention and care. A study in Australia in fitting predicted modeling for good QOL among type 2 DM patients demonstrated that knowledge on DM prevention and care was fitted to be a good predictor of good QOL [36]. Individuals with DM who have high knowledge of DM prevention and care could contribute positively their daily life by controlling blood glucose and making healthy food choice for DM patients; which could eventually lead to a better QOL.

Having complications, including diabetic nephropathy, was found to be one of the contributing factors to poor QOL among DM patients in northern Thailand. This was supported by a study conducted by Hayek et al. [37], who reported that complications of DM, such as diabetic nephropathy, were associated with poor QOL among DM patients in Saudi Arabia. A study in India also reported that type 2 DM patients with complications had a significantly poorer QOL than those who did not suffer from medical complication [39]. Didarloo et al. [28] confirmed that those with type 2 DM with complications and comorbidities had a poorer QOL than those who did not. A study in Saudi Arabia confirmed that those with type 2 diabetes mellitus without complications had a significantly better QOL than those who had complications [31]. Trikkalinou et al. also reported that the QOL among type 2 diabetes mellitus worsened when complications such as diabetes nephropathy started to develop [7]. Khunkaew et al. [25] also reported that Thai DM patients who had complications had poorer QOL. Medical complications resulting from DM could lead to several burdens and suffering of the patients and family members, which leads to poorer QOL.

A study in Spain showed that health care services, in particular their administration and cost, were defined as one of the factors influencing QOL among DM patients [4]. John et al. [39] reported that patients with type 2 DM living with poor economic status who could not afford medical fees had a poorer QOL than those who had a better economic status. In addition, a study in Nordic countries reported that free access to medical care without any barriers to medical fees and good continuity of care were associated with good QOL among type 2 diabetes mellitus patients [40]. This supported our findings that the burden of medical fees was one of the contributing factors to poor QOL among DM patients in northern Thailand. When DM is diagnosed, the patient needs to see a medical doctor regularly to check blood glucose and maintain medications to control blood glucose. This medical care often requires considerable financial commitment and becomes a barrier to attaining care, leading to poorer QOL. The burden of medical fees and barriers to maintaining a special schedule for medical checks among DM patients during the COVID-19 pandemic could have a substantial impact on QOL.

A few limitations were detected along the study. Some questions on the questionnaire asked about past experiences, such as having side effects from medicine and having experience wounds at foots, which might be a source of recall bias in the study. Due to collecting data by direct interview, some participants had difficulty identifying certain medical conditions such as hypertension and diabetic nephropathy. Therefore, another answer option, “do not know”, was created. To access full medical records of all participants is not possible, and this issue of patient recall and poor medical identification might interfere with the findings. However, the proportion of “do not know” responses was small; therefore, this might not have had much impact on the final interpretation (Additional file 2).

## Conclusions

Almost a half of DM patients in northern Thailand live with a poor QOL, especially in the physical and mental domains. However, due in part to the culture of northern Thai people, they fortunate to have a good QOL in the social relationship and environmental domains. To improve the QOL among DM patients, implementations should be emphasized to improve socioeconomic status, encourage healthier life practices, increase knowledge of DM prevention and care, and support medical fees. The schedule of monitoring blood glucose is highly correlated with the effectiveness of the program as well.

## Supplementary Information

**Additional file 1: Appendix S1**. Questionnaire.

**Additional file 2: Appendix S2**. Data.

## Abbreviations

CI: Confident interval
COVID-19: Coronavirus disease 2019
DM: Diabetes mellitus
HT: Hypertension
IOC: Item-objective congruence
IRB: Institutional review board
NCDs: Noncommunicable diseases
OR: Odds ratio
AOR: Adjusted odds ratio
QOL: Quality of life
SD: Standard deviation
WHOQOL-BREF: World Health Organization quality of life brief version

## References

[1] World Health Organization (WHO). WHOQOL: Measuring quality of life. https://www.who.int/tools/whoqol. Assessed 5 July 2021.

[2] World Health Organization (WHO). Global report on diabetes. https://apps.who.int/iris/bitstream/handle/10665/204871/9789241565257_eng.pdf;jsessionid=CB147D6BAA638DF5F8E50B368C7DFD6D?sequence=1. Assessed 5 July 2021.

[3] Jessie N, Leticia MA, Luisa AF, Alejandro GG, Gloria NA, Nelly CG (2018). Health and quality of life outcomes impairment of quality of life in type 2 diabetes mellitus: a cross-sectional study. Health Qual Life Outcomes.

[4] Elshazly HM, Hegazy NN (2017). Socioeconomic determinants affecting the quality of life among diabetic and hypertensive patients in a rural area. Egypt J Fam Med Prim Care.

[5] Ayman MEM, Ahmad M, Ashraf K, Rehab SM, Amira AF, Heba AF (2017). Impact of health education on quality of life in Egyptian type 2 diabetic patients. Int J Adv Res.

[6] Komaratat C, Auemaneekul N, Kittipichai W (2021). Quality of life for type II diabetes mellitus patients in a suburban tertiary hospital in Thailand. J Health Res.

[7] Trikkalinou A, Papazafiropoulou AK, Melidonis A (2017). Type 2 diabetes and quality of life. World J Diabetes.

[8] Degu H, Wondimagegnehu A, Yifru YM, Belachew A (2019). Is health related quality of life influenced by diabetic neuropathic pain among type II diabetes mellitus patients in Ethiopia?. PLoS ONE.

[9] Costa S, Leite A, Pinheiro M, Pedras S, Pereira MG (2020). Burden and quality of life in caregivers of patients with amputated diabetic foots. PsyCh J.

[10] Papier K, Jordan S, Este C, Banwell C, Yiengprugsawan V, Seubsman SA (2017). Social demography of transitional dietary patterns in Thailand: prospective evidence from the Thai cohort study. Nutrients.

[11] Wee SM, Henry CJ (2020). Reducing the glycemic impact of carbohydrates on foods and meals: strategies for the food industry and consumers with special focus on Asia. Compr Rev Food Sci Food Saf.

[12] Chanpiwat P, Kim KW (2019). Arsenic health risk assessment related to rice consumption behaviors in adults living in Northern Thailand. Environ Monit Assess.

[13] Bubpa N, Nuntaboot K (2018). Diversity of foods among older people in northern communities of Thailand: ways to promote health and wellness. J Health Res.

[14] World Health Organization (WHO). Coronavirus disease 2019 (COVID-19) WHO Thailand Situation Report—25 March 2020. https://www.who.int/docs/default-source/searo/thailand/2020-03-25-tha-sitrep-32-covid19-final.pdf?sfvrsn=adce58ef_0. Assessed 5 July 2021.

[15] World Health Organization (WHO). New normal non-communicable diseases clinic for healthcare workers. https://www.who.int/thailand/news/feature-stories/detail/innovative-ways-to-care-for-ncd-patients-in-thailand-in-covid-19-time-th. Assessed 5 July 2021.

[16] Charan J, Biswas T (2013). How to calculate sample size for different study designs in medical research?. Indian J Psychol Med.

[17] American Diabetes Association (2013). Standards of medical care in diabetes–2013. Diabetes Care.

[18] Department of Mental Health. Stress test-5. https://www.dmh.go.th/test/download/view.asp?id=18. Assessed 5 July 2021

[19] World Health Organization (WHO). The world health quality of life (WHOQOL). https://www.who.int/publications/i/item/WHO-HIS-HSI-Rev.2012.03. Assessed 5 July 2021.

[20] World Health Organization (WHO). The world health quality of life (WHOQOL)-Thai. https://www.who.int/substance_abuse/research_tools/en/thai_whoqol.pdf. Assessed 5 July 2021.

[21] Lima LR, Funghetto SS, Volpe CR, Santos WS, Funezl MI, Sival MM (2018). Quality of life and tine sine diagnosis of DM among the elderly. Rev Bras Geriatr Gerontol.

[22] Godman B, Shimwela M, Habte D (2018). Health-related quality of life and associated factors among patients with diabetes mellitus in Botswana. Alex J Med.

[23] Amelia R, Lelo A, Lindarto D, Mutiara E (2018). Quality of life and glycemic profile of type 2 diabetes mellitus patients of Indonesian: a descriptive study. Earth Environ Sci.

[24] Co MA, Tan LS, Tai ES, Griva K, Amir M, Chong KJ (2015). Factors associated with psychological distress, behavioral impact and health related quality of life among patients with type 2 diabetes mellitus. J Diabetes Complicat.

[25] Khunkaew S, Fernandez T, Sim J (2019). Demographic and clinical predictors of health related quality of life among people with type 2 diabetes mellitus living in northern Thailand: a cross-sectional study. Health Qual Life Outcomes.

[26] Prajapati VB, Blake R, Acharya LD, Seshadri S (2017). Assessment of quality of life in type II diabetes patients using the modified diabetes quality of life (MDQOL) questionnaire. Braz J Pharm Sci.

[27] Abedini MR, Bijari B, Miri Z, Emampour FS, Abbasi A (2020). The quality of life of the patients with diabetes type 2 using EQ-5D-5 L in Birjand. Health Qual Life Outcomes.

[28] Didarloo A, Alizadech M (2016). Health-related quality of life and its determinants among women with diabetes mellitus. A cross-sectional analysis. Nurs Midwifery Stud.

[29] Tietjen AK, Ghandour R, Mikki N, Jerden L, Eriksson JW, Norberg M (2021). Quality of life of type 2 diabetes mellitus patients in Ramallah and al-Bireh Governorate-Palestine: a part of the Palestinian diabetes complications and control study (PDCC). Qual Life Res.

[30] Mngomezulu N, Yang CC (2015). Quality of life and its correlates in diabetic outpatients in Swaziland. Int Health.

[31] Alshayban D, Joseph R (2020). Health-related quality of life among patients with type 2 diabetes mellitus in Eastern province, Saudi Arabia: a cross-sectional study. PLoS ONE.

[32] Komaratat C, Auenmaneekul N, Kittipichai W (2021). Quality of life for type II diabetes mellitus patients in a suburban tertiary hospiral in Thailand. J Health Res.

[33] Cai H, Li G, Zhang P, Xu D, Chen L (2017). Effect of exercise on the quality of life in type 2 diabetes mellitus; a systematic review. Qual Life Res.

[34] Kueh YC, Morris T, Borkoles E, Shee H (2015). Modelling of diabetes knowledge, attitudes, self-management, and quality of life: a cross-sectional study with an Australian sample. Health Qual Life Outcomes.

[35] Tapehsari BS, Alizadeh M, Khameseh M, Seifouri S, Nojomi M (2020). Physical activity and quality of life in people with type 2 diabetes mellitus: a randomized controlled trial. Int J Prev Med.

[36] Colberg SR, Sigal RJ, Fernhall B, Regensteiner JG, Blissmer BJ (2010). Exercise and type 2 diabetes. Diabetes Care.

[37] Hayek AA, Robert AA, Saeed AA, Alzaid AA, Sabaan FS (2014). Factors associated with health-related quality of life among Saudi patients with type 2 diabetes mellitus: a cross-sectional survey. Diabetes Metab J.

[38] Elazhary H, Noorwali A, Zaidi N, Alshamrani R, Aljohani M, Khan D (2018). Knowledge about diabtest its effect on quality of life among diabetic patients in King Abdulaziz University Hospital. Jeddah J Adv Med Med Res.

[39] John R, Pise S, Chaudhari L, Deshpande PR (2019). Evaluation of quality of life in type 2 diabetes mellitus patients using quality of life instrument for Indian diabetes patients: a cross-sectional study. J Midlife Health.

[40] Wandell PE (2005). Quality of life of patients with diabetes mellitus: an overview of research in primary health care in the Nordic countries. Scand J Prim Health Care.

