# Supplementary material for: Quality of life and factors associated with a good quality of life among diabetes mellitus patients in northern Thailand
Source: Health Qual Life Outcomes. 2022 May 20;20:81. doi: 10.1186/s12955-022-01986-y (PMC9122079; doi:10.1186/s12955-022-01986-y)
Supplement: Supplementary file 1 — Additional file 1: Appendix S1. Questionnaire. [file 12955_2022_1986_MOESM1_ESM.docx]

**Questionnaire**

**Blood glucose □ Controlled □ Uncontrolled**

**Part 1 P**hysical examination and biomarkers

Weight ........................................... kg Height......................................... cm

Blood pressure.................................. mmHg

LDL-Cholesterol…………………………….…. mg/dL

HDL-Cholesterol………………………………. mg/dL

Triglyceride………………………………………. mg/dL HbA1C…………………………………………………mg%

**Part 2** General information

1. Sex □ Male □ Female

2. Age…………....……. years

3. Religion □ Buddhism □ Christian □ Islam

4. Ethnic □ Thai □ Hill tribe

5. Thai ID card □ No □ Yes

6. Education □ Non-education □ Primary school □ High school □ University degree

7. Occupation □ Unemployed □ Agriculturist □ Unemployed

8. Income (per years) ..............................................Baht

9. Debt □ Yes □ No

10. Marital status □ Single □ Married □ Ever married

11. Living with □ Aloneอยู่คนเดียว □ Husband/Wife □ Daughter

12. Diabetic nephropathy □ Yes □ No □ Not sure

13. Hypertension □ Yes □ No □ Not sure

14. How long have you been diagnosed as diabetes____________ years

15. Have you forget taking diabetes medication last week? □ Yes □ No

16. Have you forget taking diabetes medication last month? □ Yes □ No

17. Do you have any experiences or side effects from diabetes medication? □ Yes □ No

18. How do you handle your medical expenses? □ Universal Health Coverage □ Pay yourself

19. Have you had any foot ulcers? □ Yes □ No

20. Family history of hypertension and diabetes

| **History** | **Diabetes** | | | **Hypertension** | | |
| --- | --- | --- | --- | --- | --- | --- |
|  | **Yes** | **No** | **Not sure** | **Yes** | **No** | **Not sure** |
| Father |  |  |  |  |  |  |
| Mother |  |  |  |  |  |  |
| Grandfather |  |  |  |  |  |  |
| Grandmother |  |  |  |  |  |  |

**Part 3** Health behaviors

1. Smoking □ No □ Ever □ Yes ________ years

2. Alcohol consumption □ No □ Ever □ Yes ________ years

3. Exercise □ No □ Sometime □ Everyday

5. How did you get daily food? □ Self cooking □ Buying

6. What kind of rice do you eat? □ Non-sticky rice □ Sticky rice

7. Having tea □ No □ Sometime □ Regularly

8. Having coffee □ No □ Sometime □ Regularly

**Part 4 St**ress assessment (ST-5) Please indicate your experience within two weeks

| **Feeling** | **No** | **Sometime** | **Often** | **Regularly** |
| --- | --- | --- | --- | --- |
| 1. Insomnia |  |  |  |  |
| 2. Lack of concentration |  |  |  |  |
| 3. Irritability |  |  |  |  |
| 4. Boring |  |  |  |  |
| 5. I do not feel like going out and meet people |  |  |  |  |

**Part 5** Assessment of knowledge about diabetes prevention and control

| **Items** | **True** | **False** | **Not sure** |
| --- | --- | --- | --- |
| 1. If a parent has diabetes, then will their child have diabetes in the future. |  |  |  |
| 2. Frequently urinating, weight loss, fatigue could be an early stage of diabetes |  |  |  |
| 3. People with diabetes are more likely to develop cataracts |  |  |  |
| 4. Having too much sugar is the cause of diabetes |  |  |  |
| 5. Being overweight are at risk for diabetes |  |  |  |
| 6. High blood pressure is one of the risk factors for diabetes |  |  |  |
| 7. Being more than 30 years of age is a risk factor for diabetes |  |  |  |
| 8. Dietary control can reduce the risk of diabetes development |  |  |  |
| 9. Regular physical activity can reduce the risk of diabetes development |  |  |  |
| 10. Diabetes is curable |  |  |  |

**Part 6** Assessment of attitudes towards diabetes prevention and control

| **Items** | **Agree** | **Neutral** | **Disagree** |
| --- | --- | --- | --- |
| 1. Diabetes is not a scary disease because everybody can be |  |  |  |
| 2. If we are diabetes, it will make fatigue, weak, and unable to work. |  |  |  |
| 3. The elderly is only a person effected by diabetes |  |  |  |
| 4. Eating a lot of white rice, it is not a risk factor of diabetes |  |  |  |
| 5. If we are not diabetic, it is not necessary to control weight |  |  |  |
| 6. Natural sugars which are found in fruits and vegetables, it is not related to diabetes |  |  |  |
| 7. If we do not take our regular medications prescribed, it is not impact of the treatment |  |  |  |
| 8. Obesity could lead to diabetes |  |  |  |
| 9. If parents do not have diabetes, then their offspring have no chance of diabetes |  |  |  |
| 10. If a patient can control their food intake, it is not necessary to see a doctor |  |  |  |

**Part 7** Quality of Life (WHOQOL-BREF)

| **Items** | | **Very**  **poor** | **Poor** | **Neither poor nor good** | **Good** | **Very**  **good** |
| --- | --- | --- | --- | --- | --- | --- |
| 1 | How would you rate your quality of life? |  |  |  |  |  |
|  | | | | | | |
| **Item** | | **Very**  **dissatisfied** | **Fairly**  **Dissatisfied** | **Neither satisfied nor dissatisfied** | **Satisfied** | **Very satisfied** |
| 2 | How satisfied are you with your health? |  |  |  |  |  |
| **The following questions ask about how much you have experienced certain things in the last two weeks.** | | | | | | |
| **Items** | | **Not**  **at all** | **A**  **Small amount** | **A**  **Moderate amount** | **A**  **great deal** | **An**  **Extreme amount** |
| 3 | To what extent do you feel that physical pain prevents you from doing what you need to do? |  |  |  |  |  |
| 4 | How much do you need any medical treatment to function in your daily life? |  |  |  |  |  |
| 5 | How much do you enjoy life? |  |  |  |  |  |
| 6 | To what extent do you feel your life to be meaningful? |  |  |  |  |  |

| **Items** | | **Not at all** | **Slightly** | **Moderately** | **Very** | **Extremely** |
| --- | --- | --- | --- | --- | --- | --- |
| 7 | How well are you able to concentrate? |  |  |  |  |  |
| 8 | How safe do you feel in your daily life? |  |  |  |  |  |
| 9 | How healthy is your physical environment? |  |  |  |  |  |

| **Items** | | **Not at all** | **Slightly** | **Somewhat** | **To a great extent** | **Completely** |
| --- | --- | --- | --- | --- | --- | --- |
| 10 | Do you have enough energy for everyday life? |  |  |  |  |  |
| 11 | Are you able to accept your bodily appearance? |  |  |  |  |  |
| 12 | Have you enough money to meet your needs? |  |  |  |  |  |
| 13 | How available to you is the information you need in your daily life? |  |  |  |  |  |
| 14 | To what extent do you have the opportunity for leisure activities? |  |  |  |  |  |

| **Item** | | **Not at all** | **Slightly** | | **Moderately** | | **Very** | **Extremely** | |
| --- | --- | --- | --- | --- | --- | --- | --- | --- | --- |
| 15 | How well are you able to get around physically? |  |  | |  | |  |  | |
| **The following questions ask you to say how good or satisfied you have felt about various aspects of your life over the over the last two weeks.** | | | | | | | | | |
| **Items** | | Very Dissatisfied | | Fairly  Dissatisfied | | Neither  Satified nor Dissatisfied | | Satisfied | Very satisfied |
| 16 | How satisfied are you with your sleep? |  | |  | |  | |  |  |
| 17 | How satisfied are you with your ability to perform your daily living activities? |  | |  | |  | |  |  |
| 18 | How satisfied are you with your capacity for work |  | |  | |  | |  |  |
| 19 | How satisfied are you with yourself? |  | |  | |  | |  |  |
| 20 | How satisfied are you with your personal relationships? |  | |  | |  | |  |  |
| 21 | How satisfied are you with your sex life? |  | |  | |  | |  |  |
| 22 | How satisfied are you with the support you get from your friends? |  | |  | |  | |  |  |
| 23 | How satisfied are you with the conditions of your living place? |  | |  | |  | |  |  |
| 24 | How satisfied are you with your access to health services? |  | |  | |  | |  |  |
| 25 | How satisfied are you with your transport? |  | |  | |  | |  |  |

| **The following question refers to how often you have felt or experienced certain things in the last two weeks.** | | | | | | |
| --- | --- | --- | --- | --- | --- | --- |
| **Items** | | **Never** | **Infrequently** | **Sometimes** | **Frequently** | **Always** |
| 26 | How often do you have negative feelings such as blue mood, despair, anxiety or depression? |  |  |  |  |  |
